# Supplementary material for: Are carbon emissions trading and green financial instruments synergistic? -Comprehensive quantitative research based on content analysis
Source: PLoS One. 2024 Mar 7;19(3):e0298601. doi: 10.1371/journal.pone.0298601 (PMC10919601; doi:10.1371/journal.pone.0298601)
Supplement: S1 Data — (ZIP) [file pone.0298601.s001.zip › Data/地级市Z_F工作报告.zip!/▒▒╛⌐/└┤╫╘╣1⁄2╓┌║┼í╛┬φ┐╦╩2╛▌═°í┐_2006.docx]

各位代表：

　　现在，我代表北京市人民政府，向大会作关于国民经济和社会发展第十一个五年规划纲要的报告，请各位代表连同《纲要(草案)》一并审议，并请市政协各位委员提出意见。

　　一、“十五”时期的回顾

　　过去五年，全市人民在党中央、国务院和中共北京市委的领导下，以邓小平理论和“三个代表”重要思想为指导，认真落实科学发展观，紧紧围绕“新北京、新奥运”战略构想，创新体制，调整结构，优化环境，加强管理，全面发展，改革开放和现代化建设取得巨大成就。

　　(一)首都经济持续快速协调健康发展，综合经济实力跃上新台阶。初步预计，“十五”期间，地区生产总值年均增长11.8%，2005年人均地区生产总值突破5000美元。经济结构不断优化，第三产业所占比重达到67.7%。累计完成全社会固定资产投资10830亿元，实现社会消费品零售额11650亿元，分别比“九五”时期增长98%和71%。坚决贯彻落实中央宏观调控政策措施，加强经济运行调节，有效抑制了经济运行中的不健康因素。地方财政收入从2000年的345亿元提高到2005年的919.2亿元，同口径年均增长25.3%；万元地区生产总值能耗、水耗年均分别降低5.5%和13%，经济增长的质量、效益明显提高。

　　(二)科技、教育、卫生稳步发展，社会事业全面进步。首都区域创新体系建设取得重大进展，中关村科技园区“五年上台阶”主要任务完成，高新技术产业集群初步形成。全市研发经费年均增长19.5%，科技进步对经济增长的贡献率不断提高。推进教育结构调整，基础教育特别是农村义务教育得到加强，高等教育率先进入普及化阶段，职业技能培训加快发展。来京务工人员子女义务教育问题基本解决。地方财政用于教育事业的支出依法增长。成功战胜“非典”疫情，有效控制高致病性禽流感疫情，加强公共卫生体系建设，大力发展基层卫生事业，城乡社区卫生服务网络覆盖率达到81%。计划生育工作稳步开展。建成一批重大公共文化设施，基层文化更加活跃，广播影视、新闻出版和哲学社会科学事业更加繁荣。全民健身运动广泛开展，北京体育健儿在雅典奥运会和全国十运会上取得优异成绩。

　　(三)城市建设步伐加快，服务能力显著提升。累计完成基础设施投资2200亿元，比“九五”时期增长60%。制定实施《北京交通发展纲要》，优化结构，挖掘存量，着力缓解交通拥堵，轨道交通建设全面提速，路网系统更趋完善。首都机场扩建、南水北调北京段工程等重点项目开工建设。能源结构得到改善，2005年用电量达到560亿千瓦时，天然气使用量31亿立方米，集中供热面积3.1亿平方米。坚持不懈地推进污染治理和生态建设，市区空气质量二级和好于二级天数达到64.1%，城八区和郊区城镇污水处理率分别达到70%和40%，城八区和郊区生活垃圾无害化处理率分别达到95.2%和46.6%，林木覆盖率、绿化覆盖率进一步提高。积极推进城市环境建设，治理城市河湖，改造公厕，整治“城中村”，市容市貌不断改善。制定实施历史文化名城保护规划和条例，加大文物保护和修缮力度，世界文化遗产得到积极保护。全面排查、坚决消除各类安全隐患，制定突发公共事件总体应急预案，建立市区两级应急指挥系统，公共安全管理显著加强。

　　(四)着力解决“三农”问题，农民利益得到有效保护。政府工作重点向农村延伸，部门联动，政策集成，大幅增加郊区投入。全面实施“221”行动计划，大力发展都市型现代农业。改善郊区基础设施状况，所有行政村铺通了柏油路，33个中心镇实现集中供水，100万农民饮水安全问题得到解决，广播电视基本实现“村村通”。完成农村药品供应和监管“两网”建设。重新启动山区搬迁工程，1.4万农民搬出泥石流易发区和采空区。深化农村改革，基本完成农村土地承包经营权确权工作，免征农业税及其附加，修订建设征地补偿安置办法，妥善解决拖欠农民征地补偿款等遗留问题。落实粮食生产直补政策，建立山区生态林补偿机制，提高生态林建设占地补偿和养护补助标准，对村级组织发展公益事业给予专项补助，农民得到更多实惠。

　　(五)改革开放深入推进，发展环境不断优化。认真贯彻《行政许可法》，调整水务等7个部门的管理体制，推进城市管理体制改革，政府职能转变取得新进展。完善了市与区县财政管理体制，财政支出结构不断优化，公共支出占财政支出的比重达到87%。国有企业改革全面推进，市属金融企业实施战略性调整，基础设施投融资改革更加深入，房地产市场管理逐步规范。文化体制改革取得重要进展。进一步精简行政审批和企业年检事项，下放审批权限。电子政务框架基本形成，全程办事代理制、“一站式”办公得到推广。不断放宽市场准入，完善扶持政策，私营个体经济长足发展。积极做好对口支援，加强与周边省区市合作。全市累计实际利用外资123亿美元，地区进出口总额3927.4亿美元，接待入境游客1460万人次。新增6个国际友好城市，成功举办《财富》论坛、诺贝尔奖获得者论坛等大型国际活动，对外交往不断深入。

　　(六)城乡居民收入大幅增加，人民生活达到新水平。城市居民人均可支配收入年均实际增长10.4%，农民人均纯收入年均实际增长9.9%，2005年分别达到17650元和7850元。实施积极就业政策，大力促进失业人员和农村富余劳动力就业，城镇登记失业率控制在2.3%以内。初步形成了覆盖城乡的社会保障体系，城镇社会保险覆盖范围稳步扩大，新型农村合作医疗加快发展，农村养老保险积极推进，社会救助体系逐步完善。城八区改造危旧房560万平方米，城镇居民人均住房使用面积达到19.5平方米，农民居住环境得到改善。消费结构加快升级，人民生活向更加宽裕的小康迈进。

　　(七)社会主义精神文明建设取得新进展，民主法制建设进一步加强。深入学习“三个代表”重要思想，认真开展保持共产党员先进性教育活动。在抗击“非典”等各项工作中，首都人民表现出高尚的精神风貌。积极开展公民道德教育实践活动，加强未成年人思想道德建设和大学生思想政治教育工作，开展新童谣征集推广活动。国防教育深入普及，双拥共建广泛开展，征兵工作和国防后备力量建设得到加强。民族、宗教、侨务和对台工作继续推进。妇女儿童、老龄、残疾人事业健康发展。

　　坚决执行人大及其常委会决议，坚持重大事项向人大常委会报告制度和向政协通报制度，充分听取人民群众对政府工作的意见和建议。制定了本市国家机关进一步接受监督的意见，自觉接受法律监督、民主监督和社会监督。认真办理人大代表、政协委员的议案、建议、提案。深入开展“四五”普法教育，健全法律服务体系。切实推进政府法制建设，五年共提请市人大常委会审议通过地方性法规和法规性决定68项，制定政府规章96项。深入开展社会治安综合治理，积极预防、控制、打击违法犯罪活动，完善人民内部矛盾纠纷排查调处工作机制，确保了首都安全稳定。

　　各位代表，“十五”期间，北京申奥取得成功，实现了中华民族的百年梦想，为首都发展注入了强大动力，奥运场馆及相关设施建设稳步推进，各项筹备工作顺利进行；完成城市总体规划修编，国务院给予肯定并做出重要批复，确定了到2020年的建设蓝图，为加快建设现代化国际城市奠定了重要基础。

　　所有这些成绩的取得，是党中央、国务院正确领导，全市人民团结奋斗的结果，凝聚着各方面的心血和智慧。我代表市政府，向全市各族人民，向人大代表、政协委员、各民主党派、工商联和无党派人士、各人民团体、社会各界人士，向中央在京单位、人民解放军和武警部队以及各兄弟省、区、市，向所有关心支持首都建设的香港特别行政区同胞、澳门特别行政区同胞、台湾同胞、海外侨胞和国际友人，表示崇高的敬意和衷心的感谢！

　　各位代表，五年来的工作实践，使我们加深了对北京发展、建设和管理客观规律的认识，提高了“四个服务”的能力和水平，积累了新的经验。

　　第一，必须注重统筹协调发展，创出首都发展新路。要毫不动摇地坚持以经济建设为中心，把握大局，紧抓机遇，加快发展。同时，要切实加强经济社会发展中的薄弱环节，努力解决“三农”问题，推动各项社会事业全面进步，实现城乡协调和可持续发展。只有这样，才能解决好为谁发展、怎样发展的问题，真正体现发展这个硬道理。

　　第二，必须着力推进增长方式转变，实现又快又好发展。无论是经济发展，还是社会建设，都要充分发挥首都资源优势，注重结构调整和增长方式转变，注重速度与结构、质量、效益相统一，增强自主创新能力，走内涵式、集约型发展道路，逐步解决首都的人口资源环境问题。

　　第三，必须加快体制机制创新，不断优化发展环境。改革是发展的强大动力。只有坚持社会主义市场经济的改革方向，加快改革步伐，才能消除制约发展的种种障碍，激发旺盛的发展活力。要充分发挥市场配置资源的基础性作用，切实转变政府职能，努力为各类市场主体创造良好的发展环境。

　　第四，必须切实强化城市管理，努力建设宜居城市。管理是城市发展的永恒主题，是实现全面协调可持续发展的重要基础。管理就是资源，管理就是效益。要寓管理于服务之中，创新管理体制、模式和手段，营造安全、便捷、舒适的城市运行环境，不断提高城市管理与服务水平。

　　第五，必须维护好人民群众的切身利益，构建社会主义和谐社会首善之区。要牢固树立“群众利益无小事”的观念，把实现好、维护好、发展好人民群众的根本利益作为一切工作的出发点和落脚点，着力解决关系人民群众切身利益的现实问题，使广大群众共享改革发展成果。

　　回顾过去的五年，我们也清醒地认识到，前进中还存在不少突出矛盾和问题，主要是：粗放型经济增长方式还没有根本转变，自主创新能力不强，经济社会发展与人口资源环境的矛盾十分突出，“十五”计划人口规模调控和空气质量改善目标远未实现；解决“三农”问题、促进城乡和区域协调发展任务相当繁重；生产安全和社会公共安全形势依然严峻，交通拥堵和环境脏乱的治理任务艰巨，“重建轻管”倾向尚未根本扭转；影响发展的体制机制问题亟待解决，转变政府职能、优化发展环境还需要下更大气力；就业压力逐步显现，收入分配矛盾较多，社会保障存在差距，正确处理改革、发展、稳定关系的难度增加，构建和谐社会首善之区任重道远。对上述问题，我们将高度重视，立足当前，着眼长远，在改革和发展中采取更加有效措施，努力加以解决。

　　二、“十一五”时期的国民经济和社会发展

　　“十一五”时期，是我们抓住机遇，实现“新北京、新奥运”战略构想的关键阶段。我们必须增强历史责任感，充分利用好各种有利条件，正确应对各种困难和挑战，立足科学发展，着力自主创新，完善体制机制，促进社会和谐，全面提高综合实力和“四个服务”水平，把首都现代化建设事业不断推向前进。

　　(一)指导方针和奋斗目标。

　　《纲要(草案)》是党中央提出科学发展观和构建和谐社会重大战略思想后，我们编制的第一个中长期规划。根据《中共北京市委关于制定北京市国民经济和社会发展第十一个五年规划的建议》，今后五年全市经济和社会发展的指导思想是：以邓小平理论和“三个代表”重要思想为指导，全面贯彻落实科学发展观，紧紧抓住全力办好2008年奥运会这个重要机遇，全面落实国务院对北京城市总体规划的批复，努力走出一条经济发展、生活富裕、生态良好的科学发展之路，实现“新北京、新奥运”战略构想，着力构建社会主义和谐社会首善之区。

　　未来五年，必须坚持以科学发展观统领经济社会发展全局，切实把首都的发展转到以人为本、全面协调可持续发展的轨道上来。为此，《纲要(草案)》提出了以下基本原则，即：坚持“首都经济”发展战略，努力保持经济平稳较快发展；坚持国家首都、国际城市、文化名城、宜居城市的发展定位，努力提高城市建设和管理水平；坚持以创新为动力，努力建设创新型城市；坚持统筹兼顾，努力促进城乡和区域协调发展；坚持经济、政治、文化、社会建设四位一体，努力构建和谐社会首善之区；坚持奥运带动战略，努力提升首都工作水平。这六条原则既是对我们工作经验和教训的深刻总结，也是在北京工作中落实科学发展观的具体要求。

　　《纲要(草案)》根据首都现代化建设“新三步走”战略部署，紧紧围绕“新北京、新奥运”战略构想，提出今后五年的主要奋斗目标是：建设创新、和谐、宜居新北京，办好一届有特色、高水平奥运会，努力实现经济发展水平、自主创新能力、可持续发展能力、人民生活品质和构建和谐社会首善之区的水平显著提升，奠定现代国际城市的基本构架。经济社会发展的主要预期指标是：地区生产总值年均增长9%，到2010年实现人均地区生产总值比2000年翻一番；地方财政收入年均增长12%；城市居民人均可支配收入和农民人均纯收入年均分别实际增长6%以上；城镇登记失业率控制在35%以内；万元地区生产总值能耗、水耗分别降低15%和20%。确定这些指标，综合考虑了首都发展的现实基础、支撑条件、发展趋势等因素，既体现一定的速度与质量要求，又适当留有余地，经过努力是可以实现的。

　　(二)推进产业结构调整和增长方式转变，走高端产业发展之路。

　　从提高产业层次和技术水平、提高规模和集聚效应、提高资源节约水平和利用效率入手，加快经济结构调整和增长方式转变，推进产业优化升级。

　　加快发展现代服务业。不断优化首都金融发展环境，积极支持金融机构推进产品和服务创新，着力推动产权交易和风险资本市场发展。制定支持文化创意产业发展的地方法规和优惠政策，打破行业垄断，鼓励资源重组，强化资金扶持，重点发展六大文化创意产业。抓住奥运契机，打造世界一流旅游城市和国际会展之都。以“稳步发展、优化结构、稳定价格”为目标，引导房地产业健康发展。改造提升商业等传统服务业，加快物流业发展。积极发展各类社会中介服务。2010年第三产业的比重达到72%左右。

　　大力发展高新技术产业。以提升自主创新能力和整体产业竞争力为核心，支持关键技术、关键产品和重大技术标准的研发和产业化，重点发展软件、研发、电子信息、生物产业，积极培育数字电视、汽车电子、新材料、新能源等潜力产业。抓好软件产业基地、国家集成电路产业园等专业园建设，形成产业集聚发展。

　　适度发展现代制造业。围绕核心技术、名牌产品和骨干企业，完善产业链条，提高现代制造业发展水平。重点发展汽车、装备制造、石化新材料、医药等产业。培育名优品牌，积极发展都市型工业。通过技术进步、优化结构，提升建筑业整体素质和竞争力。

　　发展都市型现代农业。调整优化农业结构与布局，全面拓展农业的生产、生态和生活功能，重点发展高效生态农业和观光休闲农业。推进农业的标准化生产、产业化经营和规模化发展，着力创造满足市场多样化需求的产品，深度开发特色农产品的国内外市场。

　　引导产业合理布局。发挥市场配置资源的基础性作用，实行差别化的区域导向政策和分类评价体系，合理配置重大项目，落实好空间发展战略和区县功能定位。促进重点产业和新建项目集中发展，重点建设好中关村科技园区、北京经济技术开发区、临空经济区、商务中心区、奥林匹克中心区、金融街等六大高端产业功能区。支持区县发展一批各具特色的产业集聚区。下大力气推动高能耗、高物耗、高污染、低附加值产业逐步退出，严格控制并逐步淘汰资源开采型产业，抓好首钢搬迁及新项目建设，关闭、改造城市中心区小商品集散交易市场。

　　积极发展循环经济。作为首批国家循环经济试点城市，按照减量化、再利用、资源化的原则，以资源节约和再生利用为突破口，加快循环生产、绿色消费和综合保障三大体系建设。实施产业项目综合评价，完善项目筛选的资源利用和环境保护评价指标体系，严把项目准入关。建立健全资源消耗管理制度，加强重点行业的资源消耗管理。全面推行清洁生产，强化对冶金、化工、建材、电力等行业重点企业的污染预防和全过程控制。重点发展节能省地型住宅、节能环保型汽车、节电照明设备、节水生活用品和无害化农产品。抓好再生资源利用试点，完善回收利用体系，促进各种废旧资源再利用和产业化。加强循环经济政策法规建设，搞好循环经济示范项目，探索建立循环经济评价指标体系和统计核算制度。

　　(三)大力发展社会事业，提升首都公共服务水平。

　　统筹城乡社会事业发展，加大投入，调整存量，创新服务提供模式，加快城市优质社会公共服务资源向农村转移，着力推进基本公共服务均等化。

　　优先发展教育事业。全面实施首都教育发展战略，推动应试教育向素质教育转变，加快农村教育发展，优化教育结构，提高教育质量。强化政府对义务教育的保障责任，全市九年义务教育全部免收杂费和书本费，对困难家庭学生给予补助。调整中小学布局，全面实施中小学办学条件标准化建设；着力提高教师整体素质，实施城区教师到农村支教交流制度，促进义务教育均衡发展，继续治理教育乱收费，维护教育公平。把发展职业教育和培训作为战略重点，坚持中职和高职协调发展，推进产学结合，重视农民的技能培训，不断提高劳动者素质。适应国家和首都经济社会发展需要，优化高等教育结构和布局。努力构建终身教育体系，建设学习型城市。

　　加强医疗卫生服务。完善疾病预防控制、医疗救治、卫生执法监督和卫生信息体系，全面提高首都公共卫生服务水平。做好重大疾病特别是艾滋病、结核病等重大传染病防治工作。大力发展城乡社区卫生事业，调整医院布局，实施基层卫生服务机构标准化建设，完善鼓励医务人员到基层、到农村提供服务的政策。深化医疗卫生体制改革，健全卫生服务管理和运行机制，完善医疗机构分工协作、双向转诊的医疗服务体系。加强药品和医疗服务价格监管，有效控制医疗费用不合理增长，着力解决群众看病难、看病贵问题。进一步推动中医药事业发展。深入开展爱国卫生运动，普及健康教育。

　　繁荣文化体育事业。发挥首都文化资源优势，积极发展面向基层的公益性文化事业，创造大批优秀文艺作品，满足人民群众需求。加大对社区和农村公共文化事业的支持，注重文化设施的建设和充分利用，组织好文化下农村、进社区活动，活跃基层文化生活。坚持保护、继承与发展传统民族民间文化，扶持一批体现民族特色、首都特点和国家水准的重大文化项目和艺术院团。提高新闻出版、广播影视的质量和水平，加强文化市场和互联网的管理。促进对外文化交流，创办有国际影响力的文化活动。深化文化体制改革，加快国有文化事业单位转制，鼓励社会力量发展文化事业。推进体育改革和创新，加强公共体育设施的建设与管理，深入开展全民健身活动，提高竞技体育水平。

　　(四)统筹城乡发展，建设社会主义新农村。

　　按照生产发展、生活宽裕、乡风文明、村容整洁、管理民主的要求，充分发挥农民的主体作用，扎实推进新农村建设。

　　加快农村经济发展。进一步加强农业基础设施建设，创新农田水利建设机制，改善农业生产条件。健全现代农业“七大体系”。积极培育龙头企业，加快发展专业合作组织，不断提高农业的组织化程度。大力发展农村二三产业，重点发展休闲旅游、农产品加工等特色产业，继续扩大郊区现代流通网络，鼓励中小服务企业发展。全面落实各项惠农政策，逐步加大扶持力度，努力增加农民收入，力争到2010年农民人均纯收入达到1万元。

　　深化农村各项改革。加快乡村集体经济产权制度改革。推进土地承包经营权流转，发展多种形式的适度规模经营。完善征地制度，健全对征地农民的合理补偿机制。巩固农村税费改革成果，积极推进乡镇机构改革。改善农村金融服务，建立现代农业保险制度。制定落实水源地保护办法，促进资源开采地区产业转型，实施生态、生产和生活移民，完善生态林补偿机制，推进富民养山。

　　提升村镇建设管理水平。做好新农村建设各项规划，建立有效机制，开展试点工作，改善农村生产生活条件。实施农村安全饮水工程，实现农民饮水水质达到国家标准。加快农村道路硬化、厕所改造和清洁能源使用，推行新的垃圾收集处理模式，建设适合乡村的排水和污水处理设施。优化村镇布局，着重发展一批辐射力强的中心镇，建设一批各具特色的小城镇，不断提升郊区城镇化水平。创新农村基层社会管理体制，加强农村基层政权建设。提高农民整体素质，激发农民创造新生活的自主精神，建设新型农村社区。

　　探索建立城乡统筹的新机制。加大对农村投资的倾斜力度，在保证市本级投入依法增长的基础上，市级财政新增教育、卫生、文化、计划生育等事业经费用于农村的比例不低于70%，市政府固定资产投资用于郊区的比例不低于50%，尤其要加大对乡村和山区发展的支持力度。加强部门间的统筹协调，整合政策和资金，形成支持农村发展的合力。创新体制机制，促使优质资源向郊区转移，把专业技术人才引入农村，创出“工业反哺农业、城市支持农村”的具体途径。

　　(五)加强城市管理与建设，全面提升首都现代化水平。

　　随着首都快速发展，城市管理日益重要。今后五年，北京仍处于高速建设时期，要统筹规划、建设与管理，把管理放在更加突出的位置，建好新北京，管好新北京。

　　维护城市安全高效运行。高度重视生产安全和社会公共安全，健全统一高效的应急管理体系，完善各类预案，预防、排查、控制和消除电网、地下管网、道路桥梁等各类安全隐患，加强消防、防疫、防洪、防震等设施和队伍建设，显著增强城乡防灾、减灾、救灾能力。以静态交通为重点，以智能化为依托，提高交通管理水平。加大城市环境建设力度，重点整治城乡结合部、“城中村”、重要旅游景区和奥运场馆周边地区。深化城市管理体制综合改革，建立市区街合理分工、相互衔接、规范高效的城市管理框架。完善信息化城市管理系统，提高城市管理效能。

　　加强基础设施建设。坚持城乡统筹、适度超前、增量建设与存量改造并重，着力抓好以综合交通、能源供应、水资源保障和信息通讯为主体的基础设施建设。优先发展公共交通，轨道交通运营里程达到270公里，中心城公共交通出行比例达到40%，实现全部行政村通公共汽车。加强公路建设与改造，实现公路网覆盖全市所有村镇。合理安排电源建设，加快电网发展。积极协助建设新气源工程，推动天然气管网向新城和重点城镇发展。推进可再生能源的开发利用，搞好能源储备和应急体系建设。完成南水北调北京段工程，新建中心城5座污水处理厂。以“数字北京”为目标，大力推进宽带通信网、数字电视网和下一代互联网等信息设施建设。基本建成布局合理、功能完善、技术先进的现代化邮政网络。整体规划建设一批方便市民生活、提升城市形象的街道公用设施。

　　切实保护历史文化名城。健全历史文化名城保护的管理体制和政策体系，从整体格局、历史文化保护区、文物保护单位三个层次加强保护和监管。合理确定中心城区功能与容量，控制建设规模，降低人口密度；探索小规模、渐进式有机更新方法，加强旧城整体保护。逐步改建或拆除历史文化保护区内不符合保护控制要求的建筑，加强文物建筑的抢险修缮，重视对优秀近现代建筑的保护和合理利用。正确处理保护古都风貌、改造危旧房和维护群众利益的关系，发挥政府主导作用，采取公共财政倾斜等多种措施，稳步推进危旧房改造，不断改善居住条件。

　　扎实推进新城建设。高水准完成新城规划设计，全面启动顺义、通州、亦庄三个重点新城建设，有序推进其他新城建设。充分发挥公共投资的先导作用，突出市场运作，创新融资方式，积极探索新城建设和管理的新机制。抓好一批关键性基础设施、公共服务设施及重要带动项目，引导产业集聚，创造良好的工作和生活环境，实现滚动建设、协调发展。

　　(六)加强人口资源环境工作，努力建设宜居城市。

　　促进人口资源环境与经济社会协调发展，是建设宜居城市，实现首都可持续发展的关键。

　　全面推进人口健康发展。初步预计，2005年全市常住人口已经超过1530万，五年增加166万，对城市基础设施、资源环境承载能力提出新的挑战。要运用经济、行政、法律等有效手段，实施人口综合调控，力争2010年全市常住人口规模控制在1600万。坚持计划生育，稳定低生育水平，努力提高出生人口素质。规范户籍人口迁入政策，控制人口机械增长。从对居住场所的法治化、信息化管理入手，创新流动人口管理的思路和措施，实行常态化管理、市民化服务。制定鼓励人口向新城转移的政策措施，引导人口合理分布。探索养老服务多种模式，完善老年教育、健康服务体系。维护妇女权益，创造有利于儿童健康成长的良好社会环境。做好残疾人康复、就业和社会保障工作，加强城市无障碍设施建设。

　　建设资源节约型社会。高度重视资源约束矛盾，坚持科学严格管理，采取综合措施，建立长效机制，逐步缓解水、土地、能源等紧张状况，保障城市可持续发展。健全政策体系，制定符合实际、更加严格的节约标准，实行定额和指标管理。依靠科技进步和创新，大力推广节约资源的新技术、新工艺、新设备和新材料。发展节水农业，推动再生水使用，中心城再生水利用率提高到50%。坚持集中紧凑发展模式，建设用地总量严格控制在3500平方公里以内。着力推动工业、建筑和交通节能，提高煤、电、油、气等能源利用效率，强化实施迎峰度夏和冬季供暖室温控制等需求侧管理措施。政府机构要带头节约，倡导节约风尚，树立全社会节约意识。

　　建设环境友好型城市。采取更加严格有力措施，控制污染物排放总量，改善能源结构，城市空气质量基本达到国家标准。开展水资源保护和各流域水环境污染防治，实现六环路以内主要河湖水体基本还清，中心城、新城和中心镇污水处理率达到90%。加强固体废物污染防治，建设一批生活垃圾和危险废物处理设施，危险废物实现安全处置，中心城和新城生活垃圾无害化处理率达到99%以上，农村地区达到80%。防治噪声和电磁辐射、放射性污染。继续抓好山区生态屏障、绿化隔离地区、绿色走廊和农田林网建设，不断扩大城市绿色空间，进一步提高绿化美化水平。全市林木覆盖率达到53%，城市绿化覆盖率达到45%。

　　(七)加强社会建设与管理，努力促进社会和谐。

　　完善社会管理体系，整合社会管理资源，健全党委领导、政府负责、社会协同、公众参与的社会管理格局。从解决人民群众最关心、最直接、最现实的利益问题入手，努力构建社会主义和谐社会首善之区。

　　千方百计做好就业工作。实施积极的就业政策，注重发展劳动密集型产业，大力开发社区公益性岗位，继续推进就业倍增计划。制定促进政策，实现30万农村劳动力转移就业。完善就业困难群体帮扶制度，做好首钢等国有企业富余职工分流安置工作，重视解决局部地区失业率较高的问题。完善劳动力市场信息发布制度，引导外来劳动力有序流动。建立政府扶助、社会参与的职业技能培训机制，加大培养力度，新增50万技能人才。对城镇失业人员、农民提供免费职业技能培训。建立覆盖城乡的就业服务体系，健全劳动关系调整体制。

　　完善多层次的社会保障体系。改革基本养老金计发办法，调整最低缴费标准，改革事业单位养老保险制度，完善农村养老保险制度，城镇基本养老保险覆盖率达到95%，农村养老保险覆盖率达到60%。完成公费医疗制度向医疗保险制度并轨，逐步实现新型农村合作医疗向全市统筹过渡，城镇基本医疗保险覆盖率达到95%。完善失业、工伤、生育保险制度。增加财政的社会保障投入，多渠道筹措社会保障资金。完善社会救助体系，切实保障城乡困难群众的基本生活。支持慈善事业发展，充分发挥社会捐赠、群众互助在扶贫帮困中的重要作用，推动社会福利事业社会化进程。

　　着力构建和谐社区、和谐村镇。理顺政府与城乡基层自治组织的关系，切实减轻基层自治组织行政性负担。加强基层自治组织建设，健全社区居民会议制度、协商议事制度和听证会制度，完善村民自治机制，充分发挥城乡基层组织协调利益、化解矛盾、排忧解难的作用。建立和完善社区居委会和村委会工作经费保障机制，保证社区和村级组织正常运行。实施专项补助制度，支持城乡基层组织发展公益事业。以服务群众为重点，推进面向基层、覆盖城乡、功能完善、布局合理的公共服务体系建设。制定落实《物业管理条例》实施办法，着力解决物业纠纷。大力培育民间组织，积极引导其参与和谐社区、和谐村镇建设。

　　推进精神文明建设。加强社会主义思想道德体系建设，增强广大市民的首都意识、法律意识、公德意识，全面提升市民素质和城市文明程度。认真落实人文奥运行动计划实施意见，建立健全文明礼仪行为规范体系，抓好窗口行业的奥运培训工作。加强和改进大学生思想政治工作，完善学校、家庭、社会相结合的未成年人教育网络。争创全国文明城市，塑造首都文明形象。大力普及科学知识，提高市民科学素养。深入开展国防教育和双拥共建活动，加强国防后备力量建设，创新抚恤优待等工作机制，巩固和发展军政军民关系。全面落实民族和宗教政策，制定并实施少数民族乡村经济发展规划，逐步解决宗教房产问题。切实做好侨务工作。

　　加强民主法制建设。各级政府要坚决执行人大决议，自觉接受人大监督，认真听取政协及各民主党派、工商联、无党派人士和专家学者的意见，广泛征集人民建议。充分发挥工会、共青团、妇联等人民团体的桥梁纽带作用。发展基层民主，推广厂务公开、村务公开、政务公开，扩大公民有序的政治参与。大力推进依法治市，加强行政立法工作，完善行政执法监督，广泛开展普法教育，做好法律援助，努力营造良好的法制环境。

　　全力维护首都安全稳定。切实加强对敌斗争，严密防范、坚决打击境内外敌对势力、民族分裂势力、宗教极端势力、暴力恐怖势力和“法轮功”等邪教组织的渗透、颠覆、分裂和破坏活动。健全社会治安防控体系，注重科技强警，严厉打击各种违法犯罪活动。深入研究群体性事件发生的特点和规律，积极防范、妥善处置各类群体性事件。强化信息安全工作。全力做好奥运会、庆祝新中国成立60周年等重大活动的安全保卫工作。拓宽社情民意反映渠道，落实信访工作责任制，做好基层人民调解、司法调解、行政调解工作。

　　(八)抓紧实施首都创新战略，努力建设创新型城市。

　　以中关村科技园区为核心，以重点领域和关键技术为突破口，着力提高自主创新能力，率先进入创新型城市行列。

　　强化中关村科技园区的创新带动作用。制定实施《中关村科技园区总体规划》，优化产业空间布局。完善园区技术创新体系，力争在一些重点领域取得关键技术突破，实现园区自主创新的重大跨越。实施知识产权、标准、品牌三大战略，引导企业创制行业技术标准，抢占战略制高点。深化各项综合改革，重点推进投融资体系建设，建立创业投资引导资金，支持创业投资企业发展，开展非上市股份公司股权转让试点，支持具有自主创新能力、成长性好的企业上市融资。做好为中央在京企业、科研机构和高等院校的服务工作，积极争取国家重大科技项目落户中关村。加快园区科技基础设施和公共服务配套设施建设，优化园区创新环境。

　　加快首都区域创新体系建设。深化科技体制改革，加快科研院所转制和创新主体培育，构建以企业为主体、市场为导向、产学研结合的技术创新体系。支持企业建立专业性的公共技术平台，加大研发投入，力争全市研发经费支出占地区生产总值的比重达到6%。创新政府科技投入体制，从需求出发确定科技发展方向，集中力量实施一批重大科技攻关工程，提高原始创新、集成创新和引进消化吸收再创新能力。完善和落实税收、金融、政府采购等政策措施，支持自主创新以及创新成果产业化。着力推进产权改革和股权激励试点，激发各类主体创新活力。加强各类专业孵化器建设，大力发展科技中介服务组织，建设统一的技术交易、企业融资、信息交流平台。整体规划知识产权保护工作，推进国家知识产权制度示范园区建设，强化知识产权保护的司法和执法工作，依法严厉打击各种侵犯知识产权行为，营造有利于自主创新的社会环境。

　　加强创新型人才培养。实施首都人才战略，以高层次人才为重点，加强高级管理人才、高级专业技术人才和国际化人才队伍建设，大力提高各类人才的学习、实践和创新能力。做好人才规划，创新人才资源开发与管理体制，健全人才培养、评价、选拔和使用的有效机制，推进人才资源市场化配置，引导人才合理流动。实行人才分类管理，完善社会化职称评审制度。加强留学人员创业园区建设，积极引进拥有自主知识产权、掌握尖端技术的专业人才，构筑极具活力、充满希望的人才之都，为建设创新型城市提供强大的人才保障。

　　(九)深化体制改革，不断提高对外开放和区域合作水平。

　　从解决困扰发展的矛盾入手，推进体制机制创新，扩大开放，为落实科学发展观提供可靠保障。

　　深化行政管理体制改革。以转变政府职能为重点，减少和规范行政审批，积极稳妥地推进事业单位分类改革，促进传统行业协会转型和新型行业协会发展，加快政府与所办经济实体脱钩。从体制、机制、职能、流程等方面创新政府管理，加强专家咨询、社会公示和听证、决策执行评估等制度建设，运用多种信息化手段，促进部门资源整合、信息共享、业务协同和政务公开。建立健全和严格执行问责制，提高政府执行力和公信力。全面落实投资项目核准制和备案制，大力实施非经营性政府投资项目的代建制。完善公共财政体系，优化财政支出结构，加强预算管理，提高财政资金使用效率，确保政府履行职责。强化政府对基础设施和公用事业的规划及监管职能，进一步放开建设和运营市场。积极推进资源价格改革，理顺公用事业价格，逐步建立能够充分反映市场供求状况和资源稀缺程度的价格形成机制。

　　激发市场主体活力。完善国有资本有进有退、合理流动机制，推动国有资本向重要行业和关键领域集中，向大型和特大型企业集中。健全国有资本经营预算、企业经营业绩考核和重大决策失误责任追究制度，探索建立国有非经营性资产监管体制，逐步将事业单位国有资产纳入监管范围。规范国有企业改制和产权转让，基本完成市属大中型企业的股份制改造，完善重要骨干企业法人治理结构。实现民营企业与其他企业平等待遇，鼓励非公有资本进入公用事业、基础设施和社会事业领域，参与国有企业改组改造。建立中小企业创业投资引导基金，拓宽中小企业融资渠道，培育创业辅导、人才培训等社会化服务体系。

　　推进市场体系建设。继续发展资本、技术和劳动力等要素市场。坚持并完善经营性用地“招拍挂”制度，依法规范土地市场。建设社会信用体系，培育和发展信用服务中介机构，规范信息采集、使用和管理，建立信用评价体系和管理制度。加强社会诚信宣传教育，营造诚实守信的社会环境。修订有关市场准入、行为监管及保护消费者权益的法规规章，构建行政执法、行业自律、舆论监督和群众参与相结合的市场监管体系。坚决打击制假售假、商业欺诈等违法行为。以食品、药品安全为重点，加快国家强制管理产品质量检测体系建设。

　　全面提高对外开放水平。加快转变贸易增长方式，鼓励进口先进设备和短缺资源，促进加工贸易转型升级，扩大具有自主知识产权、自主品牌的产品出口，2010年高新技术产品出口占地区出口比重达到38%。增强对反倾销等贸易争端的处置能力，指导企业维护合法权益。积极承接国际服务业转移，提高服务贸易层次和水平。引导外资投向，注重引进先进技术、管理经验和高素质人才。吸引跨国公司、大型企业在京设立地区总部、分支机构、研发中心和结算中心。完善对境外投资的协调机制和风险管理，支持有条件的企业“走出去”。进一步密切与港澳台地区的经贸交流与合作。

　　促进区域协调发展。按照国家京津冀都市圈区域开发的整体部署，发挥首都科技创新和研发中心集聚优势，抓住天津滨海新区建设机遇，建立交流合作的长效机制。加快京津城际铁路及第二高速通道建设，加强电子信息、汽车、旅游、物流等产业合作。搞好与周边地区在水资源、能源开发领域的合作，支持水源上游地区污染治理项目和环保监测项目建设。积极参与西部开发，继续做好对口支援工作。

　　(十)成功举办一届有特色、高水平的奥运会。

　　奥运战略贯穿“十一五”规划实施的全过程，我们将紧紧把握奥运机遇，努力实践“绿色奥运、科技奥运、人文奥运”理念，大力促进首都经济社会发展，全面提升各项工作水平。筹办奥运是落实科学发展观的重大实践工程。按照“节俭办奥运”的方针，举全市之力，以饱满的热情、扎实的工作、一流的业绩，完成好奥运会和残奥会的各项筹备工作，建设“阳光工程”。奥运会举办期间，精心组织好开幕式、闭幕式等大型活动，保证城市各项功能正常运转，提供一流的交通、食宿、医疗卫生、安全保卫服务和组织保障。奥运会后，注重奥运场馆的赛后利用，做好奥运品牌的持续、深度开发，保持首都经济社会的平稳健康发展。

　　三、全面完成2006年的各项任务，努力实现“十一五”规划的良好开端

　　2006年是“十一五”时期的第一年，开好局，起好步，意义十分重大。要坚持以科学发展观统领全局，努力保持首都发展的良好势头。全市经济社会发展主要预期指标是：地区生产总值增长9%，万元地区生产总值能耗、水耗分别下降2%和5%，地方财政收入增长12%，城镇登记失业率控制在25%以内，城市居民人均可支配收入和农民人均纯收入分别实际增长6%以上，居民消费价格指数调控在102%以内。突出抓好以下几个方面工作：

　　第一，实现经济平稳较快发展。努力扩大消费需求，促进旅游会展、文化体育、娱乐休闲等服务型消费，积极拓展奥运商品市场，推广新型消费方式。推动传统商业街调整改造，建设便民商业设施，发展郊区连锁超市、便利店600个。保持合理的投资规模，优化政府投资结构，引导社会资金向基础设施、现代服务业、高新技术产业和现代制造业等领域转移，促进房地产投资稳定增长，竣工经济适用住房200万平方米。加快国家软件出口基地建设，扩大高新技术产品出口，大力发展服务贸易。推动产业替代和结构优化升级，成立文化创意产业发展领导小组，研究支持文化创意产业发展的优惠政策；制定《关于促进首都金融产业发展意见》的实施细则；积极稳妥地推进首钢、焦化厂等企业搬迁，探索采矿地区产业转型的有效途径。研究制定发展循环经济指导意见。搞好煤电油气运供需调节，确保首都能源安全。

　　第二，迈出新农村建设的坚实步伐。制定新农村建设指导意见和实施办法，编制村庄建设规划，启动新农村示范村建设。加强农村基础设施建设，新增村镇集中供水能力10万吨，解决27万农民安全饮水问题，建成6座中心镇污水处理设施，自然村通油路工程新建100公里，支持农村改厕4万户。发展节水农田30万亩。继续实施“十百千万”工程，免费培训农村中小学教师，做好城镇教师到农村支教工作。建设好农村399个社区卫生服务站、133个服务中心，实现社区卫生服务基本覆盖农村人口。完成7000名山区农民搬迁、安置任务。做好第二次全国农业普查前期准备工作。

　　第三，做好关系群众生产生活的各项工作。加强创业指导服务，落实小额担保贷款、劳务派遣、社保补贴和岗位补贴等政策，实现成功创业6000人、带动就业4万人，帮助5万名“4050”失业人员走上公益性就业岗位。在80%的行政村建立就业服务站，培训农村劳动力8万人次，帮助5万名农村劳动力实现转移就业。落实《国务院关于完善企业职工基本养老保险制度的决定》，实施农村社会养老保险制度建设的意见。全面推进扩面征缴工作，确保各项基金征缴率在95%以上。做好旧城内8000户居民危房的排险解危工作，启动城八区10片危改项目。深入开展基层安全创建与平安建设。以地下管网为重点，认真排查和消除安全隐患，加强政府监管，落实法人主体责任，有效防止重特大事故发生。采取坚强有力措施，落实烟花爆竹安全管理规定。全力办好59件直接关系群众生活的重要实事。

　　第四，全力做好奥运筹备工作。充分发挥“2008”工程建设指挥部和环境建设指挥部的组织协调作用，确保各项任务按计划推进。新建场馆完成主体结构，改扩建场馆和临建场馆加快建设，奥林匹克公园形成整体景观轮廓。推进轨道交通建设，实施交通疏堵工程。支持城乡电网改造，加快燃气热电厂、天然气管线建设。整治80个“城中村”，新建和改建公厕1445座，建设100条特色园林大街。实施第十二阶段控制大气污染措施，市区空气质量二级和好于二级天数达到65%。以《首都市民文明公约》颁布10周年为契机，创建优美环境、优良秩序、优质服务，突出抓好赛场文化建设，为2008奥运会创造良好社会氛围。

　　第五，加强政府自身建设。坚持依法行政，全面推行行政执法责任制。贯彻实施公务员法，进一步规范公务员管理，加强对公务员的监督，促进勤政廉政，建设高素质的公务员队伍。坚持“四个服务”，大力推进政府管理创新和制度创新，建立部门协调配合机制，切实提高办事效率和服务水平，着力为各类企业、科研机构、事业单位，特别是为中央在京企事业单位做好服务。抓住典型事例，整治推诿扯皮、敷衍了事、效率低下等顽症，加强督察考核，做到从严治政，赏罚分明。继续加强基础工作，做到“职责清、情况明、数字准、作风正、效率高”。健全规范有效的支出约束机制，坚决反对和制止铺张浪费，强化审计监督。坚持教育、制度、监督并重，加大反腐败的工作力度，树立“为民、务实、清廉”的政府形象。

　　各位代表！首都现代化建设的蓝图已经绘就，全市人民一定能够战胜前进道路上的各种艰难险阻，共同创造更加美好幸福的明天！让我们紧密团结在以胡锦涛同志为总书记的党中央周围，高举邓小平理论和“三个代表”重要思想伟大旗帜，全面落实科学发展观，振奋精神，扎实工作，为实现“新北京、新奥运”战略构想，圆满完成“十一五”规划的各项任务而努力奋斗！
